# Metformin Treatment Was Associated with Decreased Mortality in COVID-19 Patients with Diabetes in a Retrospective Analysis

**DOI:** 10.4269/ajtmh.20-0375

**Published:** 2020-05-21

**Authors:** Pan Luo, Lin Qiu, Yi Liu, Xiu-lan Liu, Jian-ling Zheng, Hui-ying Xue, Wen-hua Liu, Dong Liu, Juan Li

**Affiliations:** 1 Department of Pharmacy, Tongji Hospital, Tongji Medical College, Huazhong University of Science and Technology, Wuhan, China;; 2 Clinical Research Center, Tongji Hospital, Tongji Medical College, Huazhong University of Science and Technology, Wuhan, China

## Abstract

Metformin was proposed to be a candidate for host-directed therapy for COVID-19. However, its efficacy remains to be validated. In this study, we compared the outcome of metformin users and nonusers in hospitalized COVID-19 patients with diabetes. Hospitalized diabetic patients with confirmed COVID-19 in the Tongji Hospital of Wuhan, China, from January 27, 2020 to March 24, 2020, were grouped into metformin and no-metformin groups according to the diabetic medications used. The demographics, characteristics, laboratory parameters, treatments, and clinical outcome in these patients were retrospectively assessed. A total of 283 patients (104 in the metformin and 179 in the no-metformin group) were included in this study. There were no significant differences between the two groups in gender, age, underlying diseases, clinical severity, and oxygen-support category at admission. The fasting blood glucose level of the metformin group was higher than that of the no-metformin group at admission and was under effective control in both groups after admission. Other laboratory parameters at admission and treatments after admission were not different between the two groups. The length of hospital stay did not differ between the two groups (21.0 days for metformin versus 19.5 days for no metformin, *P* = 0.74). However, in-hospital mortality was significantly lower in the metformin group (3/104 (2.9%) versus 22/179 (12.3%), *P* = 0.01). Antidiabetic treatment with metformin was associated with decreased mortality compared with diabetics not receiving metformin. This retrospective analysis suggests that metformin may offer benefits in patients with COVID-19 and that further study is indicated.

## INTRODUCTION

SARS-CoV-2 can cause exaggerated and aberrant noneffective host immune responses that are associated with acute respiratory distress syndrome.^1^ In these critically ill patients infected with COVID-19, the cytokine storms mediated by overproduction of pro-inflammatory cytokines have been observed in a large population.^2^ The exaggerated immune responses lead to long-term lung damage and fibrosis, causing functional disability, reduced quality of life, and even death.^3^ For these reasons, host-directed therapies were proposed to be a promising treatment for COVID-19.

The goal of host-directed therapies is to modulate immune mechanisms that relieve exaggerated inflammation to reduce lung tissue damage.^4^ Metformin, a most commonly used medication for type 2 diabetes, was proposed to be a candidate for host-directed therapy for COVID-19 to reduce mortality.^5^ However, its efficacy remains to be validated.

In this retrospective observational study, we aimed to identify the role of metformin as a host-directed therapy in COVID-19 by comparing the outcome of metformin users and nonusers in these COVID-19 patients with diabetic complications.

## METHODS

### Study design and participants.

For this retrospective study, we recruited the diabetic patients with confirmed COVID-19 discharged or died from January 27, 2020 to March 24, 2020, at Tongji Hospital in Wuhan, China. All patients were anonymous. The study was approved by the Ethical Committee of Tongji Hospital, Tongji Medical College, Huazhong University of Science and Technology (No. TJ-IRB20200338).

The diagnosis procedures of COVID-19 were referred to *the Diagnosis and Treatment of Pneumonia Infected by Novel Coronavirus* issued by the National Health Commission of China. Briefly, epidemiological history or clinical symptoms are needed. Exposure history referred to any form of body contact with confirmed cases within 14 days. Clinical features include symptoms like fever, computed tomography (CT) images with signs like patchy ground-glass opacities, and laboratory examination showing decrease in both leukocytes and lymphocytes. One with exposure history can be considered as a suspected patient if any two of the clinical features show up, but only when an exposure-free patient represents all three clinical features can he/she be suspected. The suspected patients with a positive result of any nuclear acid test or IgM–IgG test will be confirmed with COVID-19. Patients with body temperature returns to normal for more than 3 days, respiratory symptoms and lung imaging improved significantly, and two consecutive negative for nuclear acid test can be discharged.

The clinical severity of patients was graded as mildly ill (clinical symptoms were mild, and no signs of pneumonia were found on CT), moderately ill (clinical features include symptoms like fever and respiratory symptoms, and CT images with signs of pneumonia), seriously ill (respiratory rate: ≥ 30 breaths/minutes; resting oxygen saturation: ≤ 93%; or PaO_2_/FiO_2_ ratio: ≤ 300 mmHg), and critically ill (respiratory failure and mechanical ventilation, shock or intensive care required) according to *the Diagnosis and Treatment of Pneumonia Infected by Novel Coronavirus* issued by the National Health Commission of China.

The exclusion criteria of this retrospective analysis were hospital stay or medication course less than 3 days, age ≥ 85 years, and lack of information about laboratory parameters at admission. A retrospective review of the characteristics of these patients was performed through the electronic medical record system, and the medications, laboratory parameters, and outcome (mortality and the hospitalization time) were monitored.

### Statistical analysis.

Statistical analysis was carried out with SPSS (IBM Corp, Armonk, NY), version 23.0. Data are presented as median and interquartile range (IQR) or as the number and percentage, as appropriate. Wilcoxon signed rank test, Fisher’s exact probability test, and chi-square test were used to compare parameters whenever appropriate. Logistic regression analysis was used in the multivariate analysis. *P* < 0.05 was considered as statistically significant.

## RESULTS

Two hundred eighty-three diabetic patients infected with COVID-19 were enrolled into this study. One hundred four patients (metformin group) received metformin alone or with other medications for at least 3 days. The remaining 179 patients (no-metformin group) received one or multiple antidiabetic drugs other than metformin. Clinical characteristics at the time of admission are shown in Table 1. Fifty-three (51.0%) and 103 (57.5%) of the metformin group and no-metformin group participants were males (*P* = 0.28), and the age in the two groups was 63.0 (55.8–68.3) and 65.0 (57.5–71.0) years (*P* = 0.06), respectively. No significant difference was found between the two groups in underlying diseases including hypertension (*P* = 0.67), coronary heart disease (*P* = 0.10), malignancies (*P* = 0.40), chronic nephrosis (*P* = 1.00), and chronic obstructive pulmonary disease (*P* = 0.09). There is also no difference in any grade of clinical severity and category of oxygen support between metformin group and no-metformin group at admission (*P* = 0.40 and *P* = 0.43).

**Table 1:** Comparison of clinical characteristics of patients between the metformin group and no-metformin group

Characteristic	Metformin group (*n* = 104)	No-metformin group (*n* = 179)	*P*-value
Age (years)	63.0 (55.8–68.3)	65.0 (57.5–71.0)	0.06
Male gender, *n* (%)	53 (51.0)	103 (57.5)	0.28
Underlying disease, *n* (%)			
Hypertension	62 (59.6)	102 (57.0)	0.67
Coronary heart disease	11 (10.6)	32 (17.9)	0.10
Malignancies	1 (1.0)	6 (3.4)	0.40
Chronic nephrosis	1 (1.0)	3 (1.7)	1.00
Chronic obstructive pulmonary disease	0 (0.0)	6 (3.4)	0.09
Clinical severity, *n* (%)			0.40
Moderately ill	27 (26.0)	39 (21.8)	
Seriously ill	75 (72.1)	132 (73.7)	
Critically ill	2 (1.9)	8 (4.5)	
Oxygen-support category, *n* (%)			0.43
Ambient air	27 (26.0)	39 (21.8)	
Noninvasive oxygen support	76 (73.1)	135 (75.4)	
Invasive ventilation	1 (1.0)	5 (2.8)	

Data are expressed as median (IQR) or number (%). *P*-values denoted the comparison between the metformin group and no-metformin Group.

On admission, as shown in Table 2, there was no difference in the white blood count (*P* = 0.55), lymphocyte count (*P* = 0.13), monocyte count (*P* = 0.55), neutrophil count (*P* = 0.50), eosinophil count (*P* = 0.31), basophil count (*P* = 0.86), platelet count (*P* = 0.05), alanine aminotransferase levels (*P* = 0.672), aspartate aminotransferase levels (*P* = 0.39), gamma-glutamyltransferase levels (*P* = 0.91), serum creatinine levels (*P* = 0.36), blood urea levels (*P* = 0.38), and C-reactive protein levels (*P* = 0.78) between two groups. However, the fasting blood glucose level of the metformin group was higher that that of the no-metformin group at admission (*P* < 0.01).

**Table 2:** Comparison of laboratory value of patients between the metformin group and no-metformin group

Laboratory parameter	Metformin group (*n* = 104)	No-metformin group (*n* = 179)	*P*-value
White blood count (×10^9^/L)	6.12 (5.12–7.20)	6.11 (5.02–7.98)	0.55
Lymphocyte count (×10^9^/L)	1.24 (0.87–1.77)	1.08 (0.69–1.55)	0.13
Monocyte count (×10^9^/L)	0.50 (0.41–0.63)	0.50 (0.36–0.64)	0.55
Neutrophil count (×10^9^/L)	4.18 (3.29–5.19)	4.24 (3.09–5.87)	0.50
Eosinophil count (×10^9^/L)	0.05 (0.01–0.11)	0.04 (0.00–0.09)	0.31
Basophil count (×10^9^/L)	0.01 (0.01–0.03)	0.01 (0.01–0.02)	0.86
Platelet count (×10^9^/L)	237 (177–314)	222 (160–274)	0.06
Alanine aminotransferase levels (U/L)	23.0 (14.5–32.5)	22.0 (15.0–33.5)	0.67
Aspartate aminotransferase levels (U/L)	23.5 (18.0–33.0)	25.0 (19.0–35.5)	0.39
Gamma-glutamyltransferase levels (U/L)	30.0 (20.0–46.3)	28.0 (19.0–50.0)	0.91
Serum creatinine levels (umol/L)	69.0 (57.0–85.0)	71.0 (56.0–90.0)	0.36
Blood urea levels (mmol/L)	4.95 (4.00–6.00)	5.10 (3.65–7.20)	0.38
C-reactive protein levels (mg/L)	20.7 (3.40–68.2)	20.9 (2.62–83.6)	0.78
Fasting blood glucose levels (mmol/L)	9.19 (6.83–14.8)	7.36 (6.10–11.8)	< 0.01

Data are expressed as median (IQR). *P*-values denoted the comparison between the metformin group and no-metformin group.

All patients received antiviral, appropriate supportive therapies and strict glucose control after admission. As shown in Table 3, there was no difference in the use of insulins (*P* = 0.20), glucosidase inhibitors (*P* = 0.31), insulin secreting drugs (*P* = 0.12), dipeptidyl peptidase-4 inhibitors (*P* = 0.65), and insulin-sensitizing agents (*P* = 0.33) between two groups. The use of antiviral including arbidol (*P* = 0.45), lopinavir–ritonavir (*P* = 0.11), chloroquine/hydroxychloroquine (*P* = 0.62), ribavirin (*P* = 0.38), interferon (*P* = 0.60), and Chinese traditional medicine (*P* = 0.11) such as Lianhua Qingwen Capsules did not differ between the two groups. No significant difference was found between two groups in antibacterial treatment (*P* = 0.99), anticoagulant therapy (*P* = 0.11), and hormonotherapy (*P* = 0.72). The use of statins, another promising agent for host-directed therapy, was also not different between the two groups (*P* = 0.95). Multivariate analysis showed that the use of metformin (*P* = 0.02) and Chinese traditional medicine (*P* = 0.02) was negatively correlated with the in-hospital mortality of patients (Table 4).

**Table 3:** Comparison of treatment of patients between the metformin group and no-metformin group

Treatment	Metformin group (*n* = 104)	No-metformin group (*n* = 179)	*P*-value
Antidiabetic treatment, *n* (%)			
Insulins	61 (58.7%)	91 (50.8%)	0.20
Glucosidase inhibitors	53 (51.0%)	80 (44.7%)	0.31
Insulin secreting drugs	28 (26.9%)	34 (19.0%)	0.12
Dipeptidyl peptidase-4 inhibitors	11 (10.6%)	16 (8.9%)	0.65
Insulin sensitizing agents	6 (5.8%)	6 (3.4%)	0.33
Antiviral treatment, *n* (%)			
Arbidol	77 (74.0%)	125 (69.8%)	0.45
Lopinavir–ritonavir	25 (24.0%)	29 (16.2%)	0.11
Chloroquine/hydroxychloroquine	8 (7.7%)	11 (6.1%)	0.62
Ribavirin	12 (11.5%)	15 (8.4%)	0.38
Interferon	10 (9.6%)	14 (7.8%)	0.60
Chinese traditional medicine	79 (76.0%)	120 (67.0%)	0.11
Antibacterial treatment, *n* (%)	72 (69.2%)	124 (69.3%)	0.99
Anticoagulants, *n* (%)	26 (25.0%)	61 (34.1%)	0.11
Glucocorticoids, *n* (%)	40 (38.5%)	65 (36.3%)	0.72
Statins, *n* (%)	20 (19.2%)	35 (19.6%)	0.95

Data are expressed as number (%). *P*-values denoted the comparison between the metformin group and no-metformin group.

**Table 4:** Multivariate analysis of the relation between in-hospital mortality and treatment in COVID-19

Treatment	Multivariate analysis	
	Odds ratio (95% CI)	*P*-value
Metformin	4.36 (1.22–15.59)	0.02
Statins	2.98 (0.65–13.76)	0.16
Arbidol	1.51 (0.60–3.84)	0.38
Lopinavir–ritonavir	1.10 (0.33–3.63)	0.88
Chloroquine/hydroxychloroquine	1.48 (0.17–12.53)	0.72
Ribavirin	0.43 (0.12–1.51)	0.19
Interferon	0.47 (0.13–1.66)	0.24
Chinese traditional medicine	3.02 (1.22–7.51)	0.02

Although no significant difference was found in the hospital stays between two groups (*P* = 0.74), the in-hospital mortality of 2.9% (3/104) in the metformin group was markedly decreased compared with the mortality of 12.3% (22/179) in the no-metformin group (*P* = 0.01) (Table 5).

**Table 5:** Comparison of clinical outcome of patients between the metformin group and no-metformin group

Clinical outcome	Metformin group (*n* = 104)	No-metformin group (*n* = 179)	*P*-value
Hospitalization time (days)	21.0 (15.0–28.0)	19.5 (12.0–26.3)	0.74
In-hospital mortality, *n* (%)	3 (2.9%)	22 (12.3%)	0.01

Data are expressed as median (IQR) or number (%). *P*-values denoted the comparison between the metformin group and no-metformin group.

## DISCUSSION

It is apparent that diabetes will increase the risk of SARS-CoV-2 infection and can worsen the outcome of this new coronavirus disease.^6^ Given the many metabolic similarities, including hyperglycemia, higher levels of pro-inflammatory cytokines, and oxidative stress, between these two diseases, these facts may help to identify candidate host-directed therapy targets for COVID-19 timely.^7–9^ Therefore, it would be reasonable to expect that the most frequently prescribed medication for diabetes, metformin, may be a candidate host-directed therapy for COVID-19.^5^ In this retrospective study, we evaluated the beneficial effect of metformin in COVID-19 patients with diabetes. The data showed that antidiabetic treatment with metformin appears to be associated with a decreased mortality in COVID-19 patients. And surprisingly, multivariate analysis suggested that application of Chinese traditional medicine may also have a potential benefit in reducing mortality in COVID-19 patients.

There has been accumulation of evidence pertaining to the underlying mechanisms of metformin as a potential agent in host-directed therapy. Metformin has been shown to improve the immune response and reduce inflammation by promoting the formation of M2 macrophages and T-regulatory and CD8 memory T cells.^10^ It also reduces the expression of genes encoding cytokines and chemokines associated with inflammation response.^11^ Moreover, metformin has been found to be benefit for microbiota composition and consequently reduce inflammation.^12^ In addition, metformin use can induce autophagy, which has a role in killing or containing pathogens, controlling inflammation, and activating innate and adaptive immune response in the host.^13^ Furthermore, metformin can stimulate adenosine monophosphate–activated protein kinase activity, then enhance protection against oxidative stress,^14^ and change the activities of catalase and superoxide dismutase.^15^ These roles suggest that metformin may be beneficial for COVID-19 control.

Although the fasting blood glucose level of patients in the metformin group was higher than that in the no-metformin group at admission, it was under effective control in both groups after admission. There was no difference of the clinical characteristics, other laboratory parameters, and concurrent medications in metformin users and nonusers at admission. These suggest that the condition was comparable between the two groups. Moreover, our results showed that the in-hospital mortality of metformin users was lower than that of nonusers in COVID-19 patients with diabetes. However, there was no difference in hospital stays between two groups. This is most probably because the primary goal of host-directed therapies is to modulate immune mechanisms that diminish excess inflammation to prevent the transition from the very first symptoms to acute respiratory distress syndrome (a life-threatening lung condition) in COVID-19 patients. However, effects of host-directed therapies on SARS-CoV-2 are likely very limited. Therefore, treating with metformin is expected to have little impact on viral clearance or length of hospital stay when discharge is premised on negative viral nucleic acid tests (../../AppData/Roaming/Program Files (×86)/Youdao/Dict/7.5.2.0/resultui/dict/discharge of patients).

Metformin’s glucose-lowering effect is achieved by enhancing the activity of existing insulin and reducing hepatic glucose production. For this reason, it is well tolerated and does not usually cause hypoglycemia in diabetic or nondiabetic patients. Moreover, metformin has a low risk of lactic acidosis in patients with altered liver or kidney function. Therefore, metformin therapy is ideally suited for repurposing as host-directed therapies for COVID-19 patients whether they have diabetes or not.

In conclusion, this retrospective study suggests that metformin may contribute to reduce the mortality due to COVID-19 and justifies the implementation of a randomized clinical study in hospitalized nondiabetic patients with COVID-19.
